# Investigating the High-Temperature Water/MgCl_2_ Interface through Ambient Pressure Soft X-ray Absorption Spectroscopy

**DOI:** 10.1021/acsami.3c02985

**Published:** 2023-05-18

**Authors:** Francesco Tavani, Matteo Busato, Daniele Veclani, Luca Braglia, Silvia Mauri, Piero Torelli, Paola D’Angelo

**Affiliations:** † Dipartimento di Chimica, Università di Roma “La Sapienza”, P.le A. Moro 5, 00185 Roma, Italy; ‡ Istituto per la Sintesi Organica e la Fotoreattività (ISOF), Consiglio Nazionale delle Ricerche (CNR), via P. Gobetti 101, 40129 Bologna, Italy; § CNR - Istituto Officina dei Materiali, TASC, I-34149 Trieste, Italy; ∥ Dipartimento di Fisica, Università di Trieste, Via A. Valerio 2, 34127 Trieste, Italy

**Keywords:** XAS, NEXAFS, soft-XAS, MCR analysis, MgCl_2_, Water/MgCl_2_ interface

## Abstract

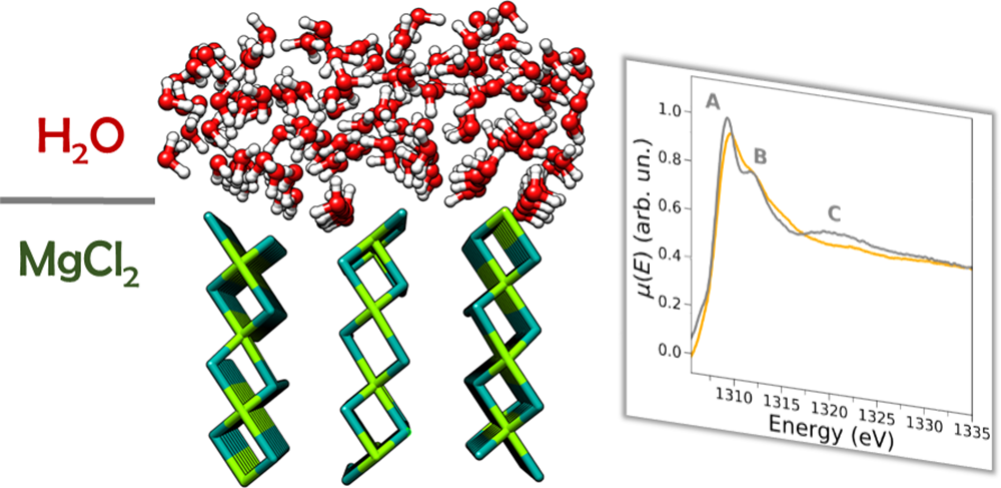

Magnesium chloride is a prototypical deliquescent material whose surface properties, although central for Ziegler–Natta cataysis, have so far remained elusive to experimental characterization. In this work, we use surface-selective X-ray absorption spectroscopy (XAS) at ambient pressure in combination with multivariate curve resolution, molecular dynamics, and XAS theoretical methods to track in real time and accurately describe the interaction between water vapor and the MgCl_2_ surface. By exposing MgCl_2_ to water vapor at temperatures between 595 and 391 K, we show that water is preferentially adsorbed on five-coordinated Mg^2+^ sites in an octahedral configuration, confirming previous theoretical predictions, and find that MgCl_2_ is capable of retaining a significant amount of adsorbed water even under prolonged heating to 595 K. As a consequence, our work provides first experimental insights into the unique surface affinity of MgCl_2_ for atmospheric water. The developed technique is proven highly sensitive to the modifications induced by adsorbates on a given low-Z metal based surface and may be useful in the toolbox required to disentangle the mechanisms of interfacial chemical processes.

## Introduction

Surface aqueous interfaces are ubiquitous in natural and technological processes. Gaining deep chemical knowledge on the mechanisms of water interaction with surfaces is paramount to addressing challenging questions in atmospheric and planetary sciences, geochemistry, and catalysis. Here, temperature plays an important role in tuning the orientation of the water interfacial layers and the surface interaction energies. Surfaces in fact possess unique temperature-dependent properties that may significantly differ from those of the bulk and act as efficient energy-dissipating and symmetry-altering heat baths toward water gas phase molecules in both reactive and nonreactive conditions. Consequently, innovative experimental and theoretical methods are required to shed light onto the often elusive electronic and structural modifications induced by water on a given surface at temperatures that may be far from ambient ones.

Deliquescent salts are salts that can absorb sufficient quantities of water vapor to form an aqueous solution in which they are fully dissolved.^1^ Deliquescence occurs at a humidity termed deliquescence relative humidity (DRH), which is temperature dependent^1,2^ and is a property exhibited by hygroscopic chloride salts relevant to atmospheric and planetary sciences, such as NaCl, CaCl_2_, and MgCl_2_. Na^+^, Mg^2+^, and Cl^–^ ions are the most abundant ionic constituents of seawater, and it has been suggested that NaCl–MgCl_2_ mixture particles may play a key role in atmospheric chemistry by acting as nascent sea-spray aerosol (SSA) surrogates and nucleating centers for cloud formation, as opposed to pure NaCl particles.^3−8^ It is important to observe that, while SSAs deviate from pure salt particles and constitute complex systems of high organic composition, the detailed chemical mechanisms of cloud condensation from SSAs and the role played by the surface in stabilizing water vapor interaction remain poorly understood.^9^

Herein, we resort to a surface-specific advanced experimental spectroscopic technique to quantitatively investigate the interface between water vapor and a prototypical hygroscopic chloride salt at *T* > 390 K, choosing MgCl_2_ (α form) as a model system. Structurally disordered MgCl_2_ (δ form) plays a central role in the stereoselective polymerization of propylene, where it acts as the active support for Ziegler–Natta catalysts.^10^ For this reason, the adsorption properties of a number of catalytically relevant MgCl_2_ surfaces have been investigated by theoretical and experimental methods, with a particular emphasis on the coordinatively unsaturated ones. Notably, recent dispersion-corrected periodic density functional theory (DFT-D) calculations suggested that, in well-formed MgCl_2_, in the absence of adsorbates, the unsaturated (104) surface exhibiting five-coordinate Mg cations is the energetically favored one.^11−13^ The conclusion that five-coordinated Mg^2+^ cations are the dominant adsorption sites was supported by the lower surface energy of the (104) surface if compared to that of the (110) surface, where the Mg^2+^ cations are four-coordinated, a finding later confirmed by infrared spectroscopy carbonyl compound adsorption studies on both chemically activated and dry-milled MgCl_2_.^14^ However, this system is highly sensitive to the conditions in which MgCl_2_ is prepared, and DFT-D theoretical modeling has suggested that MgCl_2_ crystals preferentially expose the (110) surfaces when they are formed in the presence of silanes or small molecules such as methanol and ethanol, which act as Lewis bases.^15,16^ Moreover, despite the significant theoretical effort, a limited number of experimental techniques have been employed so far to track the MgCl_2_ surface modifications upon interaction with gas-phase molecules in realistic conditions, and spectroscopic data concerning water adsorption on MgCl_2_ surfaces are almost nonexistent.^17,18^ Consequently, it is important to employ innovative surface-specific experimental techniques to address the questions of what are the structural and electronic properties of the surface MgCl_2_ sites upon which water vapor is preferentially adsorbed in ambient pressure conditions and what is the temperature dependence of such interaction. Among the advanced experimental spectroscopic techniques that may be used to simultaneously access structural and detailed electronic information, X-ray absorption spectroscopy (XAS) stands out as a useful method to monitor the local modifications of a selected photoabsorber.^19−21^ While the use of XAS in the high-energy region is now routine,^22,23^ this technique has been less employed to investigate surfaces containing low-Z metal ions, due to the requirement of soft X-rays (∼400–2000 eV) and of special experimental set-ups.^24^ In fact, XAS beamlines operating in the soft X-ray regime (soft-XAS) need high-vacuum conditions and only very recently specific cells allowing *operando* soft-XAS measurements at ambient pressures have been made available.^25−28^ This technique, termed ambient pressure near edge X-ray fine structure spectroscopy (AP-NEXAFS), allows one to record total electron yield (TEY) soft-XAS spectra and is intrinsically surface-sensitive, since the electron escape depth from the probed sample is low. In the present study, we combine *operando* soft-XAS with advanced theoretical analyses to study the MgCl_2_ surface upon its exposure to water vapor at temperatures greater than 390 K. We determined the preferential structural adsorption geometries of the water molecules on the MgCl_2_ surface, found that a prototypical hygroscopic chloride-containing surface such as MgCl_2_ may retain adsorbed water molecules at temperatures exceeding 390 K, and implemented a novel experimental method to investigate in real-time interfaces containing low-Z metal ions.

## Materials and Methods

### Experimental AP-NEXAFS Measurements

The AP-NEXAFS experiments were performed at the APE high energy beamline at the Elettra synchrotron radiation source. The AP-NEXAFS *operando* data collection was made possible by the use of a specific reaction cell, which is displayed in Figure S1 of the Supporting Information (SI). The samples inside the cell may be exposed to fluxes of different gases at a pressure of 1 bar and can be heated above 600 K. A Si_3_N_4_ membrane mounted on the top of the cell separates the volume of the reactor cell at atmospheric pressure from the ultrahigh vacuum beamline environment, while being transparent to the soft X-rays. The sample is lodged inside the reactor, perpendicular to the incoming X-rays while inlet and outlet pipes allow the gas flow inside the reactor during NEXAFS measurements. A ceramic heater located below the sample and outside the reactor allows the sample heating. The spectra are collected in TEY mode. The membrane is positively polarized, and the drain current of the sample is measured by means of two electrical contacts, one placed on the Si_3_N_4_ membrane and the other on the sample holder. A picoammeter is used to measure the drain current.

MgCl_2_ was purchased from Sigma-Aldrich. The powder was fixed on a titanium sample holder and pressed in a pit lodged onto the holder. The MgCl_2_ initial sample was pretreated at a temperature of 595 K in flowing He at 50 standard cubic centimeters per minute (SCCM). Subsequently, the sample was exposed to water vapor at 593 K with He acting as the carrier gas. The temperature was then slowly decreased to 391 K while exposing the sample to water vapor. During the experiment, the Mg K-edge spectra were collected in the range 1275–1355 eV at the temperatures and times listed in Table S1 and under a flowing gas mixture of 3% H_2_O/He at 50 SCCM and 1 bar. The time required to record every *operando* AP-NEXAFS Mg K-edge spectrum was approximately 5 min. Our experimental procedure is reported below:

1. First, a clean MgCl_2_ sample (pretreated in a He flux at 595 K to eliminate physisorbed species) was exposed to water vapor using He as a carrier gas at 593 K for ∼30 min.
2. Subsequently, the temperature was decreased up to 391 K using a −2.0 K per minute average rate while exposing the sample to water vapor.
3. Finally, the water flux was interrupted and the working temperature was increased back to 595 K with a + 2.9 K per minute average rate while exposing the sample to an inert atmosphere.

### MD Simulations

Classical molecular dynamics (MD) calculations were performed to simulate the water/MgCl_2_ interface. Surface geometries were obtained from the crystallographic structures of bulk α-MgCl_2_,^29^ by cleaving the solid in the normal (100) directions. This slab has been chosen as representative of the possible exposures of the Mg^2+^ cations on the MgCl_2_ surface, since along the (100) direction the crystal exposes a recurring sequence of 3-, 5-, and 6-coordinated magnesium centers (hereafter named Mg-3, Mg-5, and Mg-6, respectively).^30,31^ A vacuum region of about 40 Å was introduced between adjacent 8 × 12 × 4 (Mg_384_Cl_768_) supercells, so that the simulation box final dimensions were 29.1 × 70.7 × 53.0 Å^3^. The magnesium and chlorine atoms were fixed at the crystallographic positions and set out of the equations of motion. An amount of 411 water molecules were initially placed at random positions on the *xy*-plane of the MgCl_2_(100) slab to simulate the absorption of an aqueous film. A snapshot of the employed simulation box is shown in Figure S2. The nonbonded part of the interaction potential comprised a Lennard-Jones (LJ) functional form with cross-terms constructed by the Lorentz–Berthelot combining rules, plus a Coulomb potential. The partial charges for the magnesium and chlorine atoms were set equal to the nominal oxidation numbers, i.e., + 2.0 *e* and −1.0 *e*, respectively, with *e* being the elementary charge. The LJ parameters for the magnesium centers were taken from those developed by Babu and Lim to describe the coordination of the Mg^2+^ ion in aqueous solution,^32^ while those for the chlorine atoms were taken from the OPLS force field.^33^ The structure and interactions of the water molecules were described by the SPC/E model.^34^ A cutoff radius of 12 Å was employed for all the nonbonded interactions, while the long-range electrostatic forces were computed with the particle Mesh Ewald method.^35,36^ All the stretching vibrations involving the hydrogen atoms were constrained with the LINCS algorithm.^37^ The simulation protocol consisted of an energy minimization followed by 60 ns production runs in the NVT ensemble at either 413 or 593 K (*vide infra*). The first 20 ns were discarded as equilibration time. The temperature was controlled with a Nosé–Hoover thermostat,^38,39^ with a relaxation constant of 0.5 ps, while the equations of motion were integrated with the leapfrog algorithm with a time step of 1 fs and the atomic positions were saved every 100 steps. All the simulations were carried out with the Gromacs 2019 package,^40^ and the VMD 1.9.3 software was used for trajectories visualization.^41^

## Results and Discussion

**Figure 1:**
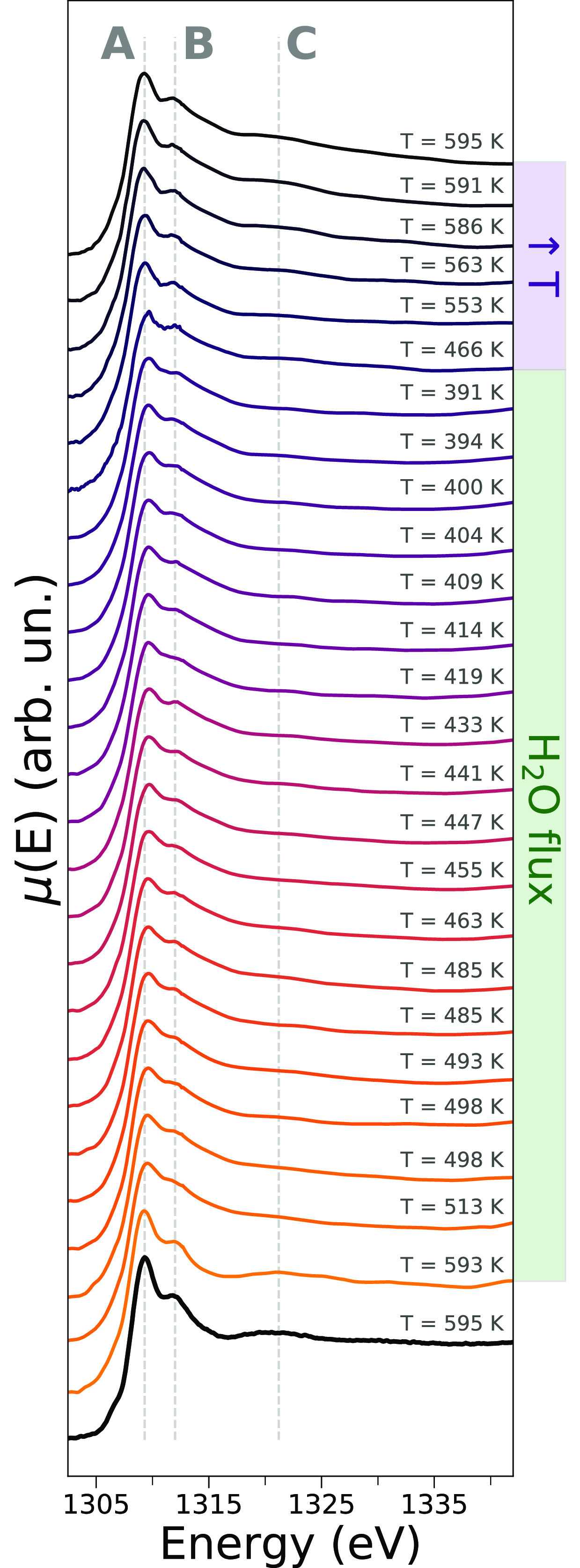
Evolution of the *operando* Mg K-edge AP-NEXAFS spectra upon exposure of MgCl_2_ to water vapor at temperatures in the 595–391 K range. Constant energy cuts are drawn at 1309.3, 1312.0, and 1321.2 eV (gray dashed lines) and are labeled as features A, B, and C, respectively. The spectra recorded during the water flux and during the subsequent flux interruption and temperature increase up to 595 K are highlighted by green and purple lateral panels, respectively. The AP-NEXAFS spectrum recorded before the surface exposure to the water flux and at the temperature of 595 K is evidenced in a bold black line.

The newly developed AP-NEXAFS technique, which is intrinsically surface sensitive, has been used in combination with an in-depth theoretical approach to study the water/MgCl_2_ interface at ambient pressure and at temperatures greater than 390 K. Figure 1 shows the Mg K-edge AP-NEXAFS spectra measured on the MgCl_2_ sample upon its exposure to water flux in the temperature range 595–391 K and after flux interruption and temperature increase again up to 595 K. The XAS spectrum recorded on the pristine MgCl_2_ sample prior to water flux and at a temperature of 595 K is evidenced by a black full line. Three distinctive transitions are present in the NEXAFS spectrum of the pristine sample that are located at 1309.3, 1312.0, and 1321.2 eV and are labeled **A**, **B**, and **C**, respectively. The intensities of such features vary appreciably during the exposure of the MgCl_2_ surface to water vapor and temperature decrease. Specifically, the intensity difference between features **A** and **B** in the normalized XAS spectra decreases, together with the intensity of feature **C**, and feature **A** also undergoes a positive energy shift of ca. 0.4 eV. Upon water flux interruption and temperature increase to 595 K, the intensity difference between features **A** and **B** partially increases again and so does the intensity of feature **C**, while feature **A** is shifted about 0.4 eV to lower energies.

**Figure 2:**
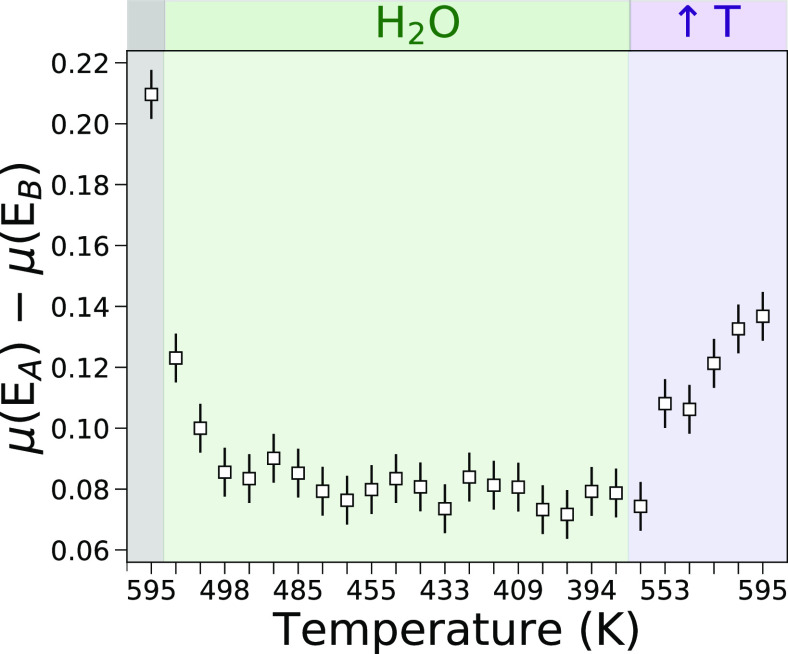
Variation of the intensity difference of the features **A** and **B** in the normalized *operando* Mg K-edge XAS spectra collected upon MgCl_2_ exposure to water vapor. The data recorded for the initial XAS spectrum, for the XAS spectra collected during water flux, and for the XAS spectra measured after flux interruption and simultaneous temperature increase are shown in gray, green, and purple backgrounds, respectively.

The evolution of the intensity difference between features **A** and **B** in the normalized *operando* Mg K-edge NEXAFS spectra (i.e., the function μ(*E*_A_) – μ(*E*_B_)) is shown in Figure 2. Looking at this figure it may be noticed that the μ(*E*_A_) – μ(*E*_B_) function reaches a maximum value in the NEXAFS spectrum of the pristine MgCl_2_ sample, while it decreases to a minimum upon water flux for temperatures between 485 and 391 K. Once the water flux is interrupted and the temperature is brought again to 595 K, the μ(*E*_A_) – μ(*E*_B_) function increases again reaching a value that is approximately half of its initial value in the pristine sample. These findings qualitatively suggest that the water vapor does interact with the Mg^2+^ ions at the MgCl_2_ surface and that such an interaction is reversible. We note that, although the employed acquisition time scheme did not allow us to observe the full reversibility of the interaction between the water vapor and the MgCl_2_ surface, it appears reasonable to expect that, a more prolonged treatment of the sample at ∼595 K, after water flux interruption, would allow for the recovery of the pristine MgCl_2_ surface, a process that also occurs during the sample pretreatment. Notably, while performing a similar AP-NEXAFS experiment to investigate the water/MgO interface, we recently observed that the water/MgO interaction is entirely reversed after treating the MgO sample up to 525 K for about 20–30 min.^22^ Conversely, the fact that in our experimental conditions the water vapor/MgCl_2_ interaction is not fully reversed after ca. 70 min of thermal treatment may be ascribed to the significantly different MgCl_2_ surface properties if compared to those of MgO, e.g., to the higher higroscopicity of the MgCl_2_ material.

In order to quantitatively determine the number of pure chemical species contributing to the total experimental NEXAFS signal, the experimental data were analyzed employing a mathematical decomposition method belonging to the multivariate curve resolution (MCR) family. In this theoretical framework, it is possible to apply the Lambert–Beer law to retrieve the spectral and concentration profiles of the key Mg^2+^ surface species contributing to the total NEXAFS measured signal.^42^ The method allows one to rationalize often complex spectroscopic XAS data sets and has been recently applied, for instance, in the mechanistic investigation of solution chemistry reactivity.^20,21,43−45^ In the first step, a statistical analysis based on the scree plot statistical test has been carried out, and the results are shown in Figure S3. Looking at this figure, one may observe that there is an elbow in the plot of the singular values as a function of the related principal components between the second and third component, thus indicating the presence of two components in the Mg K-edge NEXAFS data set.

**Figure 3:**
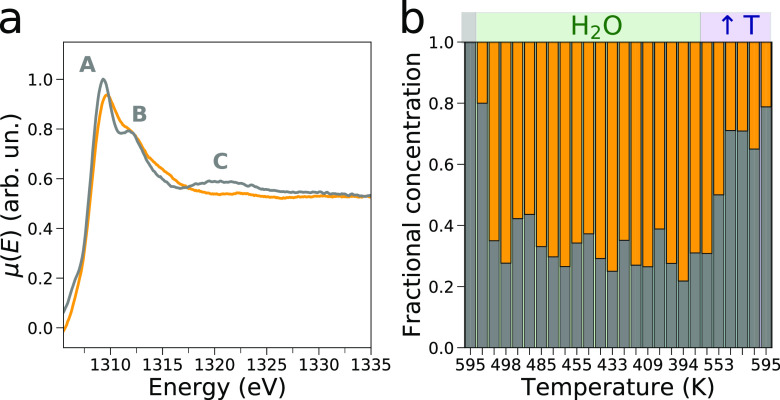
Results of the MCR analysis of the *operando* Mg K-edge XAS data. (a) Extracted NEXAFS spectra associated with the pristine MgCl_2_ spectrum (gray line) and second component (orange line). (b) Extracted concentration profiles associated with the first (gray) and second (orange) spectral components.

In the second step, the AP-NEXAFS data were analyzed by means of the MCR transition matrix decomposition approach employing a number of components equal to two (refer to the SI for additional details). The MCR-extracted concentration and Mg K-edge spectra are shown in Figure 3. In our analysis, the NEXAFS spectrum measured on the pristine MgCl_2_ sample at 595 K, which is in fair agreement with previous MgCl_2_ K-edge measurements,^46^ was constrained to coincide with the first extracted spectral component (gray curve in Figure 3). In the second extracted NEXAFS spectrum (orange curve in Figure 3), the intensity of features **B** and **C** is depleted, the quantity μ(*E*_A_) – μ(*E*_B_) decreases, and the energy of transition **A** is positively shifted of ca. 0.4 eV if compared to that of the MgCl_2_ spectrum. This second component is assigned to the Mg^2+^ intermediate species arising from the interaction of the Mg^2+^ ions on the MgCl_2_ surface and the fluxed water molecules. The fractional concentration of this spectral component reaches values close to 20% during the initial water flux at 593 K and then rapidly increases up to ∼70% once the temperature is lowered below 513 K. Interestingly, the fractional concentration of the intermediate Mg^2+^ species remains between ∼60% and ∼70% in the temperature range 513–391 K where no significant spectral changes are observed. Once the water flux is interrupted and the temperature is increased again to 595 K, the fractional concentration of MgCl_2_ becomes predominant. Notably, the NEXAFS spectrum of the intermediate Mg^2+^ species is markedly different from the XAS spectrum assigned to a fully dissolved octahedral Mg^2+^ ion.^22,47^ The body of evidence we have presented suggests that an interaction between the surface Mg^2+^ ions and the water vapor molecules is established while excluding the occurrence of a surface dissolution process.

### Overview of the Structural Arrangement at the Water/MgCl_2_ Interface: MD Results

**Figure 4:**
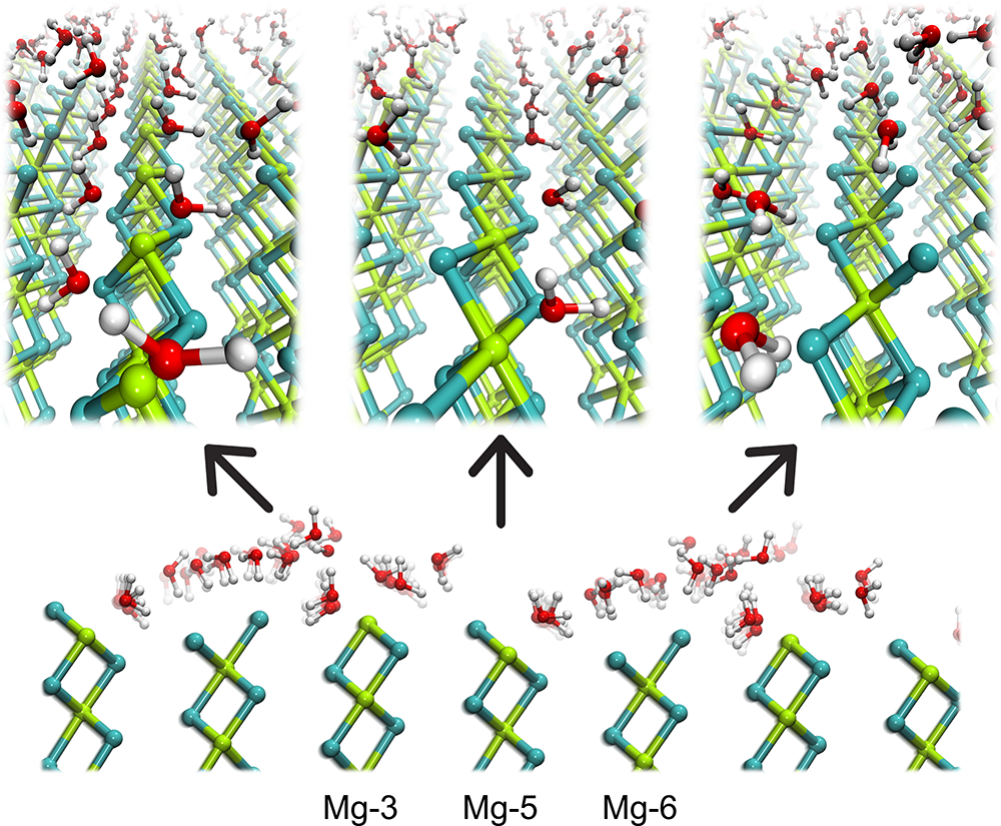
Representative snapshots taken from the final configuration of the MD simulation performed on the water/MgCl_2_(100) system at 413 K, showing the structural arrangement of the water molecules interacting with the Mg-3, Mg-5, and Mg-6 sites (green, Mg; cyan, Cl atoms).

To get insights into the structural arrangement of the water molecules at the interface, radial pair distribution functions *g*(*r*)’s between the magnesium centers of the MgCl_2_(100) surface and the oxygen atoms of the water molecules have been calculated from the MD simulations performed on the water/MgCl_2_ system. As previously mentioned, the (100) slab exposes recurring Mg-3, Mg-5, and Mg-6 sites corresponding to magnesium atoms that are unsaturated of chloride anions and thus fully accessible, partially saturated, and fully covered by chloride anions, respectively. To better characterize the individual contributions in terms of magnesium exposure, the *g*(*r*)’s have been computed for the Mg-3, Mg-5, and Mg-6 sites separately, and the obtained curves are shown in Figure S4. For both the Mg-3 (Figure S4a) and Mg-5 (Figure S4b) types, a distinct first peak is found at Mg–O distances of 2.09 and 2.20 Å, respectively. Such a contribution indicates that there is a direct interaction of the water molecules with these surface magnesium sites, with this distance also being close to the Mg–O one previously determined for the Mg^2+^ cation in aqueous solution.^22,48^ The average number of water molecules interacting with each magnesium center has been obtained by integrating the *g*(*r*)’s up to a cutoff distance chosen at the first minimum after the first peak. As a result, each Mg-3 site is surrounded by an average number of 3.0 water molecules, while the integration number for the Mg-5 sites resulted to be 1.0. Inspection of the MD snapshots shown in Figure 4 indicates that each Mg-3 site is coordinated by one water molecule set on top of the layer crest, plus two water molecules that are shared with the two contiguous Mg-3 centers. The latter two are oriented in order to point one hydrogen atom toward the chloride anions of the closest Mg-6 layer, establishing a hydrogen-bond (H-bond) interaction. In a similar way, each Mg-5 site is coordinated by one water molecule interacting by means of the oxygen atom, while at the same time directing one hydrogen atom toward the closest chloride anions on the surface of the adjacent Mg-6 layer (Figure 4). On the other hand, the *g*(*r*) calculated between the Mg-6 centers and the oxygen atom of water (Figure S4c) shows a main peak centered at 5.42 Å. As expected, no direct Mg–O interactions can be therefore established between the water molecules and the saturated Mg-6 sites, with the latter ones being fully covered by chloride anions and thus not directly accessible. Conversely, this result is compatible with a H–Cl interaction between the hydrogen atoms of the water molecules and the chloride anions of the surface. A closer look to the MD snapshots in Figure 4 reveals that these water molecules can H-bond with those interacting with the adjacent Mg-3 centers on one side and the Mg-5 ones on the other side. This arrangement is made possible since the water molecules interacting with the Mg-6 sites *via* the hydrogen atoms are free to receive a H-bond on the oxygen atom, while those interacting with the Mg-3 and Mg-5 sites *via* the oxygen atom are free to donate the H atoms for H-bonding. The whole result is evocative of a plethora of situations where the structural arrangement of the water molecules at the water/MgCl_2_ interface is strictly oriented by the shape of the crystal surface and, in this particular case, by the recurrence of the Mg-3, Mg-5, and Mg-6 layers. Also note that a similar picture is obtained at both 413 and 593 K as shown by the Mg–O *g*(*r*)’s (Figure S4), which are almost superimposable at the two tested temperatures, albeit an expected broadening of the main peaks observed for the higher temperature due to thermal disorder effects.^49,50^

### Theoretical Calculation of the NEXAFS Spectra

**Figure 5:**
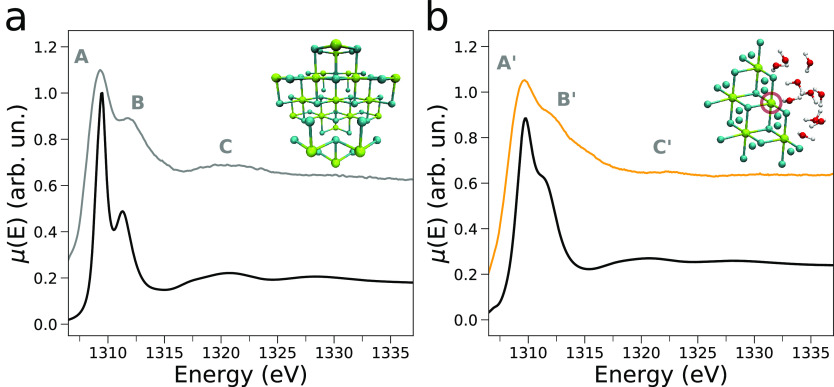
(a) Mg K-edge NEXAFS simulated spectrum of MgCl_2_ (black line), together with the experimental MCR-extracted curve (gray line) along with the associated MgCl_2_ cluster. (b) Comparison between the NEXAFS spectrum of the Mg^2+^ species arising at the MgCl_2_ surface upon its exposure to water vapor (orange line) and the average theoretical Mg K-edge NEXAFS spectrum of the Mg-5 surface site (black line). The latter theoretical curve is the average of 100 MD snapshots of the water/MgCl_2_ interface at 593 K and 100 snapshots of a MD simulation of the same system conducted at 413 K. A surface Mg-5 site directly coordinating one water molecule is evidenced with a circle in a representative cluster (green, Mg; cyan, Cl; red, O; white, H atoms).

In order to investigate the properties of the Mg^2+^ surface intermediate species, an *ab initio* DFT NEXAFS analysis was performed (see the SI for details). As a first step, the theoretical framework was tested by calculating the NEXAFS spectrum of MgCl_2_ starting from the literature crystal structure (space group *R*3*m*, *a* = 3.6363(1) Å, *c* = 17.6663(5) Å, *V* = 202.31 Å^3^).^51^ The theoretical XAS spectrum of the MgCl_2_ system is shown in Figure 5a and presents a good agreement with the experimental MgCl_2_ spectrum. In fact, both the relative intensities and energy positions of the main transitions **A**, **B**, and **C** are correctly reproduced by the calculation. Specifically, note that transition **B** at ∼1312 eV is pronounced in the calculated spectrum as in the experimental curve, even if the absolute intensities are slightly different between the theoretical and experimental data.

After having benchmarked our approach on the MgCl_2_ crystal structure, we turned our attention to uncover the nature of the Mg^2+^ surface intermediate arising during the water vapor flux. It is well-known that, for disordered systems as the water/MgCl_2_ high-temperature interface, the experimental NEXAFS signal results from the average of the coordination geometries of the water molecules with the most stable surface Mg^2+^ sites, and a single configuration alone cannot be used for a complete description of the system.^52−54^ In order to properly account for the effects of temperature and structural fluctuations, it is possible to carry out the theoretical analysis starting from the MD description of the interface. In particular, average NEXAFS theoretical spectra were calculated for clusters of Mg-5, Mg-3, and Mg-6 sites starting from 100 snapshots of MD simulations performed at 593 and 413 K, and the results are shown in Figures S5 and S6, respectively. As expected, the XAS spectra of the Mg-3 sites are the most sensitive to the configurational disorder at both temperatures due to the higher mobility of the water molecules directly coordinating the Mg^2+^ centers, and they present the most pronounced differences among each other. The differences among the spectra of the Mg-5 and Mg-6 sites are instead more limited. Figure S7 compares the theoretical NEXAFS 100-spectra averages resulting from MD simulations performed at 593 and 413 K for the Mg-3, Mg-5, and Mg-6 surface sites. In all cases, the NEXAFS spectra evaluated at the two temperatures are nearly identical. This finding together with the facts that (i) at 593 and 413 K the Mg–O *g*(*r*)’s are almost superimposable (Figure S4), (ii) two main statistical components contribute to the experimental XAS spectra, and (iii) the concentration evolution of the Mg^2+^ surface species interacting with water exhibits small changes during the vapor flux in the 593–391 K temperature range support the conclusion that water vapor is adsorbed at the surface Mg sites in essentially the same geometries at temperatures between 593 and 391 K.

**Figure 6:**
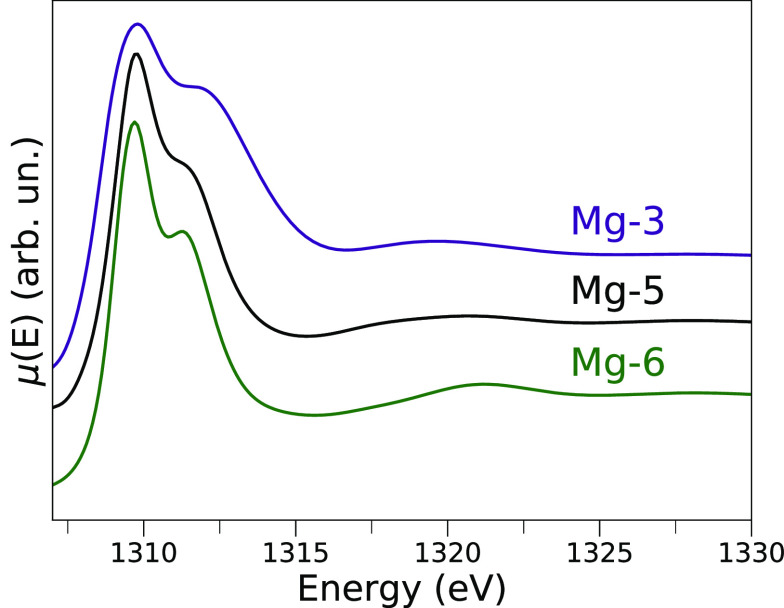
Mg K-edge average theoretical NEXAFS spectra of the Mg-3 (purple), Mg-5 (black), and Mg-6 (green) surface sites. The theoretical curves are the averages of 100 MD snapshots of the water/MgCl_2_ interface at 593 K and 100 snapshots of a MD simulation performed at 413 K.

Figure 6 shows the theoretical NEXAFS spectra of the Mg-3, Mg-5, and Mg-6 surface sites, evaluated as the averages of 100 MD snapshots at 593 and 413 K. One may note that three main transitions located at approximately 1309, 1312, and 1320 eV are present in all simulated spectra, but their relative intensity is different. In particular, in the XAS spectra of the Mg-3 and Mg-5 sites, which are directly coordinated by water molecules, the transition at ∼1312 eV is less pronounced if compared to that of the Mg-6 site. In order to investigate the origin of this behavior, we calculated the MD-averaged spectra of the Mg^2+^ sites belonging to the molecular planes below those of the surface Mg-5 and Mg-3 sites, which we term here Mg_sub_-5 and Mg_sub_-3 sites. As one may see in Figure S8, in both the NEXAFS spectra of the Mg_sub_-5 and Mg_sub_-3 sites, the transition at ∼1311 eV is enhanced and its intensity is very similar to that present in the theoretical spectrum of bulk MgCl_2_.^55^ Consequently, the intensity depletion of this feature in the theoretical spectra of the Mg-5 and Mg-3 sites is due to the direct coordination of water molecules to the Mg^2+^ centers. The XAS spectra of the Mg_sub_-3 and Mg_sub_-5 sites are instead similar to the XAS bulk signal, as expected due to their increased distance from the adsorbed water molecules. As previously mentioned, earlier studies have reported that, in clean and well-formed MgCl_2_, the (104) surface exhibiting Mg-5 cationic sites is the most stable one.^11−13^ It is important however to point out that this picture is highly sensitive to the conditions in which MgCl_2_ is prepared, whose variation may favor for instance the exposure of different sites such as the Mg-4 ones.^15,16^Figure 5b compares the theoretical average spectrum of the Mg-5 site to the experimental MCR-extracted spectrum resulting from the interaction of the surface with water vapor. The three main experimental features, and especially transition **B’**, which is less pronounced than feature **B** in pristine MgCl_2_, are nicely reproduced by the calculation. This result suggests that in our experimental conditions the (104) slab could be the most stable one exhibiting Mg-5 sites to preferentially interact with water vapor. Given however the certain degree of similarity of the theoretical NEXAFS spectra of the Mg-5, Mg-3, and Mg-6 sites (see Figure 6) and the limited sensitivity of the AP-NEXAFS technique in distinguishing only slight intensity differences in the energy region of **B’**, one cannot fully exclude the limited adsorption of water on the Mg-3, Mg-6, or possibly, Mg-4 sites. However, on the basis of the presented statistical analysis, the degree of water vapor adsorption on such differently coordinated and less energetically favored sites is not expected to exceed a few percent of that on the total surface.

## Conclusions

In this work, we have combined the advanced surface- and element-specific AP-NEXAFS technique with a multivariate and theoretical investigation to study and properly describe the nature of the interaction established by water vapor and the surface of a prototypical hygroscopic chemical system, i.e., MgCl_2_. Specifically, we leverage MCR, MD, and DFT-assisted NEXAFS analyses to show that upon controlled exposure of MgCl_2_ to vapor at temperatures between 593 and 391 K and at ambient pressure water molecules are preferentially adsorbed on five-coordinated unsaturated surface Mg^2+^ sites, which exhibit an overall octahedral geometry, in detectable concentrations. This result provides often elusive experimental confirmation of previous theoretical models predicting the favored stability of (104) MgCl_2_ surfaces, whose importance is most evident in Ziegler–Natta catalysis. Further, we experimentally demonstrate the strength of the interaction between water and the MgCl_2_ surface. We find that, at high water vapor coverages, the MgCl_2_ surface interacts with water molecules in the whole 593–391 K temperature range. Such an interaction is established by exposing the MgCl_2_ surface to the water flux already at 593 K for ∼30 min and is essentially preserved unchanged between 513 and 391 K. This second result evidences how strongly the free surface Mg^2+^ sites bind water molecules and supports the notion that hygroscopic minerals such as MgCl_2_ may capture water playing important roles in atmospheric and planetary water harvesting processes. Further, we point out that in our experimental conditions the percentage of Mg^2+^ ions dissolved at the water/MgCl_2_ interface is expected to be negligible, a behavior that differs from that of the water/MgO interface, where the dissolution of Mg^2+^ surface species at 313 K was recently found to occur.^22^ The key role played by high temperature in our experiment is thus 2-fold: on the one hand, by keeping *T* > 390 K, we exclude local Mg^2+^ dissolution; on the other hand, we exploit the elevated temperatures to probe the ability of the MgCl_2_ surface in coordinating and capturing free water molecules. In conclusion, the presence of a low-abundant intermediate at the high-temperature water/MgCl_2_ interface has been quantitatively uncovered by the AP-NEXAFS and MCR combined method. We expect this work to provide new experimental insights into the role played by low-Z metal containing hygroscopic materials and especially by MgCl_2_, in water capture through the application of soft-XAS.

## Supporting Information Available

Discussions of experimental details, MCR decomposition of the NEXAFS spectra, and details on the theoretical calculations of the NEXAFS spectra, table of temperature and time recording of each of the AP-NEXAFS Mg K-edge spectra, and figures of 3D rendering of the experimental reaction cell, snapshot of the initial MD configuration, results of the scree plot statistical test, MD Mg–O radial distribution functions of the magnesium cations on the MgCl_2_ (100) surface, and evaluation of the NEXAFS theoretical spectra calculated from MD snapshots of the water/MgCl_2_ interface (PDF)
