# Supplementary material for: Investigating the High-Temperature Water/MgCl2 Interface through Ambient Pressure Soft X-ray Absorption Spectroscopy
Source: ACS Appl Mater Interfaces. 2023 May 18;15(21):26166–74. doi: 10.1021/acsami.3c02985 (PMC10236435; doi:10.1021/acsami.3c02985)
Supplement: Supplementary file 1 — am3c02985_si_001.pdf [file am3c02985_si_001.pdf]

Supporting Information

Investigating the High Temperature Water/$MgCl_2$ Interface through Ambient Pressure Soft X-ray Absorption Spectroscopy

Francesco Tavani,[a] Matteo Busato,[a] Daniele Veclani,[b]
Luca Braglia,[c] Silvia Mauri,[c,d] Piero Torelli,[c] Paola D'Angelo[a,*]

[a] Dipartimento di Chimica, Università di Roma "La Sapienza",
P.le A. Moro 5, 00185 Roma, Italy.
[b] Istituto per la Sintesi Organica e la Fotoreattività (ISOF), Consiglio Nazionale delle Ricerche (CNR), via P. Gobetti 101, 40129 Bologna, Italy.
[c] CNR - Istituto Officina dei Materiali, TASC, I-34149 Trieste, Italy.
[d] Dipartimento di Fisica, Università di Trieste,
Via A. Valerio 2, 34127 Trieste, Italy.
[*] p.dangelo@uniroma1.it

1 Ambient Pressure Near Edge X-ray Absorption Fine Structure measurements

Table S1: Temperature and time recording of each of the AP-NEXAFS Mg K-edge spectra (shown in Figure 1 of the main text) recorded during the experiment involving the exposure of $MgCl_2$ to water.

Scan number	Temperature (K)	Time (min)
1	597(2)	0
2	593(2)	13
3	513(2)	40
4	498(2)	45
5	498(2)	50
6	493(2)	55
7	485(2)	60
8	485(2)	65
9	463(2)	70
10	455(2)	75
11	447(2)	80
12	441(2)	85
13	433(2)	90
14	419(2)	100
15	414(2)	105
16	409(2)	110
17	404(2)	115
18	400(2)	120
19	394(2)	125
20	391(2)	130
21	466(2)	140
22	553(2)	165
23	563(2)	170
24	586(2)	190
25	591(2)	195
26	595(2)	200

2 Decomposition of the AP-NEXAFS data into the spectra and fractional concentrations of key components

Time-resolved spectroscopical measurements of chemical processes yield a series of spectra that may be positioned in a matrix $\boldsymbol{D}$, where the columns of $\boldsymbol{D}$ are the spectra measured at time t. According to Lambert-Beer's law, at any given time a number N of "pure" and independent components weighed by their fractional concentration contributes to the measured signal.[1] Decomposing the experimental data into the spectra associated to the key species and in their relative concentration profiles can offer important insight in the investigated process. In the present work, such decomposition was performed with the PyFitit code,[1] a software that uses to such end an algorithm belonging to the MCR family.
The starting point is the Singular Value Decomposition (SVD) equation:

$$\boldsymbol{D} = \boldsymbol{U} \cdot \boldsymbol{\Sigma} \cdot \boldsymbol{V} + \boldsymbol{E} \tag{1}$$

where the product $\boldsymbol{U} \cdot \boldsymbol{\Sigma}$ contains, on its N columns, a set of values that may be associated to the normalized absorption coefficients, $\boldsymbol{\Sigma}$ is a diagonal matrix known as the *singular values* term, whose elements are sorted in decreasing order, while $\boldsymbol{V}$ can be interpreted as the concentration matrix associated to the N-selected components. Lastly, the error matrix $\boldsymbol{E}$ represents the lack of fit between the experimental data matrix $\boldsymbol{D}$ and the reconstructed matrix $\boldsymbol{\mu} = \boldsymbol{U} \cdot \boldsymbol{\Sigma} \cdot \boldsymbol{V}$. The SVD deconvolution depends on the correct estimation of the number of components N present in the experimental spectral matrix. To this end, in this investigation we evaluated N by performing a scree plot test, whose results are shown in Figure S3 below.
At this point, all matrices in Equation 1 are solely mathematical solutions to the decomposition problem without physico-chemical meaning. Once N is established, the approach implemented by PyFitIt requires the introduction of a transformation N $\times$ N matrix $\boldsymbol{T}$ in Equation 1, using the relation $\boldsymbol{I} = \boldsymbol{T} \cdot \boldsymbol{T}^{-1}$:

$$\boldsymbol{D} = \boldsymbol{U} \cdot \boldsymbol{\Sigma} \cdot \boldsymbol{T} \cdot \boldsymbol{T}^{-1} \cdot \boldsymbol{V} + \boldsymbol{E} \tag{2}$$

where the spectra belonging to the key species are given by $\boldsymbol{S} = \boldsymbol{U} \cdot \boldsymbol{\Sigma} \cdot \boldsymbol{T}$ and their concentration profiles by $\boldsymbol{C} = \boldsymbol{T}^{-1} \cdot \boldsymbol{V}$. Subsequently, the matrix elements T_{ij} of matrix $\boldsymbol{T}$ are modified by sliders to achieve $\boldsymbol{S}$ and $\boldsymbol{C}$ which are chemically and physically interpretable. Once this step is achieved, one can finally write:

$$\boldsymbol{D} = \boldsymbol{S} \cdot \boldsymbol{C} + \boldsymbol{E} \tag{3}$$

The unknown number of T_{ij} elements of $\boldsymbol{T}$ is in principle equal to N^2. In order to reduce such ambiguity, the AP-NEXAFS measured on the clean $MgCl_2$ surface was constrained to coincide with the first extracted spectral component. This operation allows the reduction of the number of unknown T_{ij} elements from N^2 to $N^2 - N$.

3 Theoretical NEXAFS simulations

The Mg K-edge absoption spectra presented in this study were calculated using the FDMNES code, implementing the recently developed sparse solver method.[2–4] This software is based upon the Finite Difference Method (FDM), an attractive approach for the simulation of the photoelectron wave function beyond 100 eV above the absoption edge, avoiding the muffin tin approximation used in many common multiple scattering theory based codes. Specifically, the unit cell-normalized cross section $\sigma(\omega)$ was calculated as:

$$\sigma(\omega) = 4\pi^2\alpha\hbar\omega \sum_{j}\sum_{f,g} |\Psi_f|\Theta|\Psi_g^{(j)}|^2\delta(\hbar\omega - (E - E_g^{(j)})) \tag{4}$$

where $\hbar\omega$ is the energy of the photon, α the fine structure constant, E_g and E are the energies of the ground state $\Psi_g^{(j)}$ and Ψ_f, respectively, while the summation over j includes the contribution of all the atoms in the unit cells possessing index j.[5] The electron - photon interaction is treated classically employing the operator Θ, neglecting the magnetic part of the electromagnetic field and describing its electring portion with the first two terms of the multipolar expansion (corresponding to electric dipole and electric quadrupole excitations):

$$\Theta = \boldsymbol{\epsilon} \cdot \boldsymbol{r}(1 + \frac{i}{2}\boldsymbol{k} \cdot \boldsymbol{r}) \tag{5}$$

where $\boldsymbol{r}$ is the relative position from the photoabsorber, $\boldsymbol{\epsilon}$ is the photon polarization and $\boldsymbol{k}$ the photon wave vector. In all calculations the Schrōdinger-like equation was solved self-consistently to find the final states where there is a transition.[5]

The calculated cross-sections were convoluted in a post-processing step by an energy-dependent arctangent function (Γ) in order to compare them to the experimental NEXAFS data. Γ is defined as follows:

$$\Gamma = \Gamma_i + \Gamma_f(\frac{1}{2} + \frac{1}{\pi}arctan(\frac{\pi\Gamma_f}{3E_w}(\frac{\boldsymbol{E} - E_f}{E_c} - \frac{E_c^2}{(\boldsymbol{E} - E_f)^2}))) \tag{6}$$

where $\boldsymbol{E}$ is the energy scale of the Mg K–edge NEXAFS spectrum, Γ_i and Γ_f are the core-level and final-state widths, respectively, E_c and E_w are the center and width of the arctangent function, respectively, while E_f is the Fermi energy.[6] In order to reproduce the local environment of the Mg^{2+} ion at the surface of $MgCl_2$ within a sufficient number of atomic planes, as probed by the AP-NEXAFS technique, the NEXAFS spectrum of MgO was simulated including in the calculation all scattering atoms within a cutoff radius of 6Å. The NEXAFS spectra obtained from the MD-extracted configurations of all the surface Mg sites described in the main text were simulated including in the calculations water molecules and chlorine atoms within 6 Å of the photoabsorber, i.e. until convergence was reached.

4 Supplementary Figures S1–S8

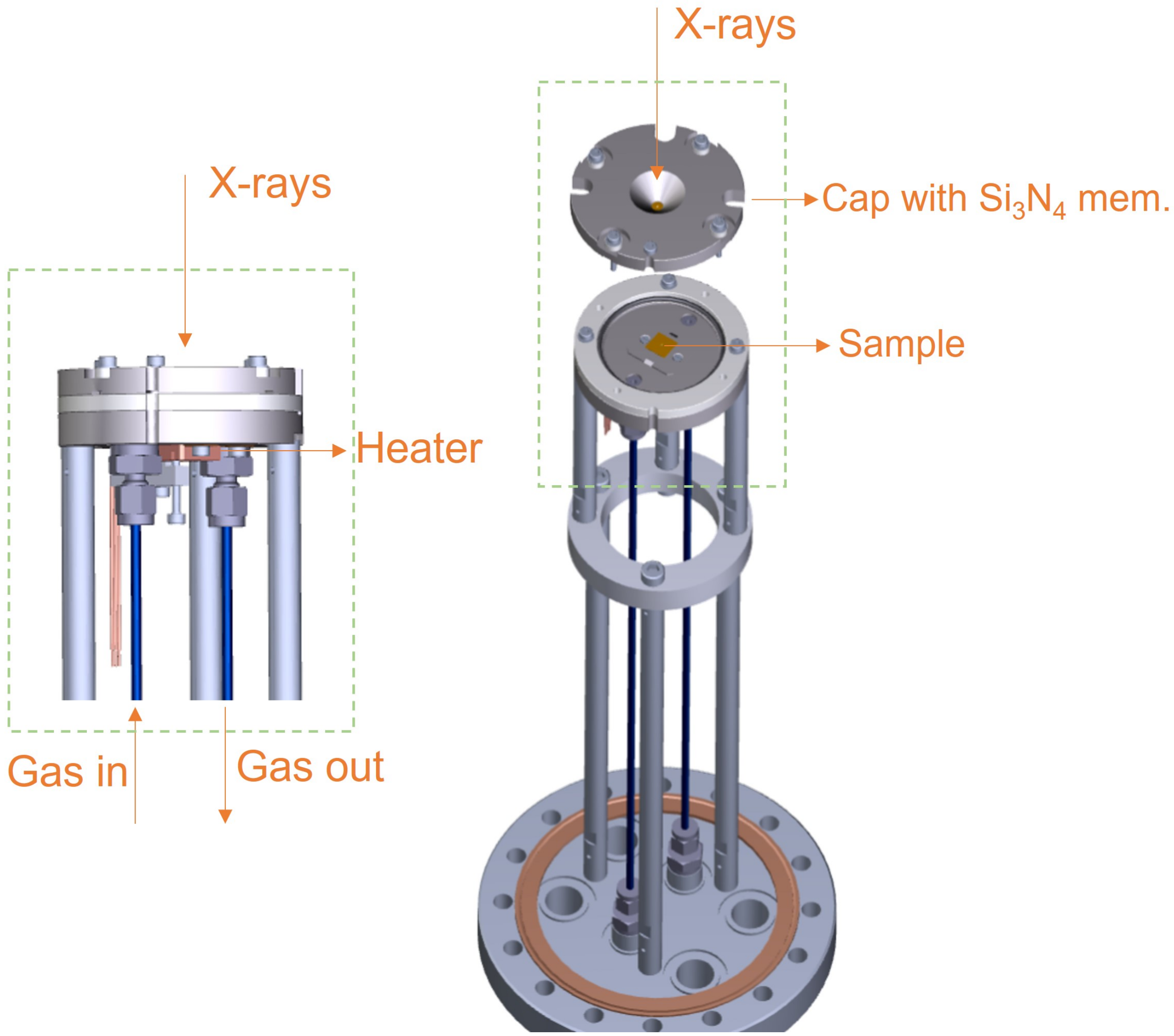

Figure S1: a) 3D rendering of the reaction cell developed at APE-HE for the *operando* NEXAFS experiment.

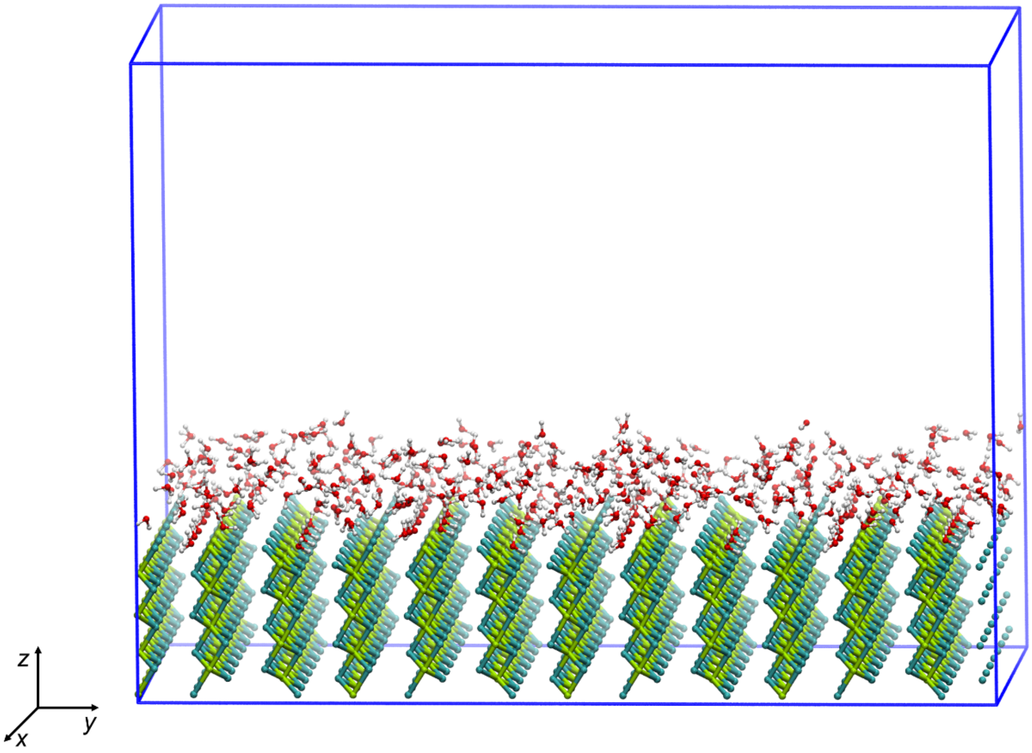

Figure S2: Snapshot taken from the initial configuration showing the box employed for the MD simulation of the $MgCl_2(100)$ surface (green: Mg, cyan: Cl atoms) with an absorbed water film. Box edges are highlighted by blue lines.

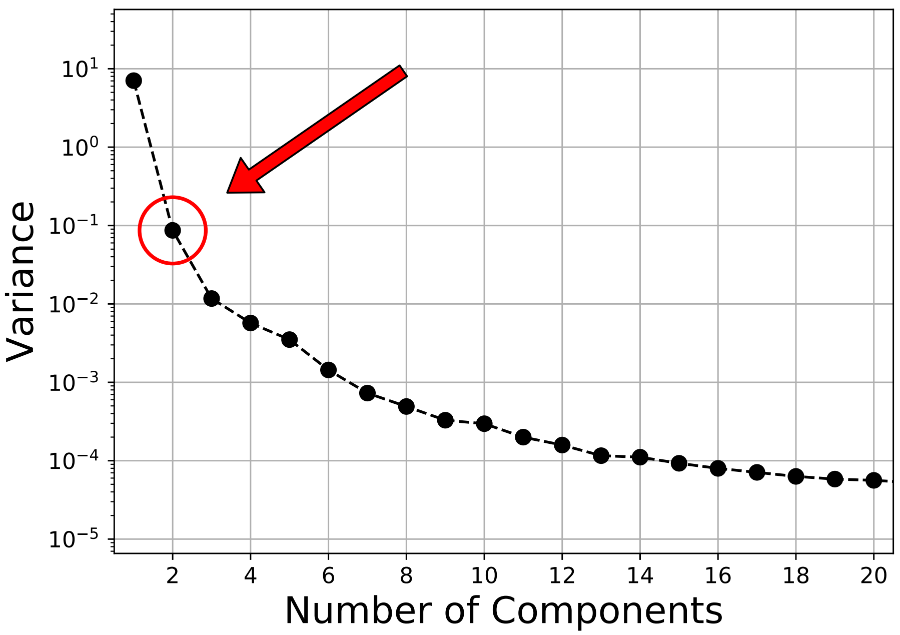

Figure S3: Scree plot statistical analysis of the Mg K–edge *operando* NEXAFS data. The test indicates the presence of N=2 components in the dataset.

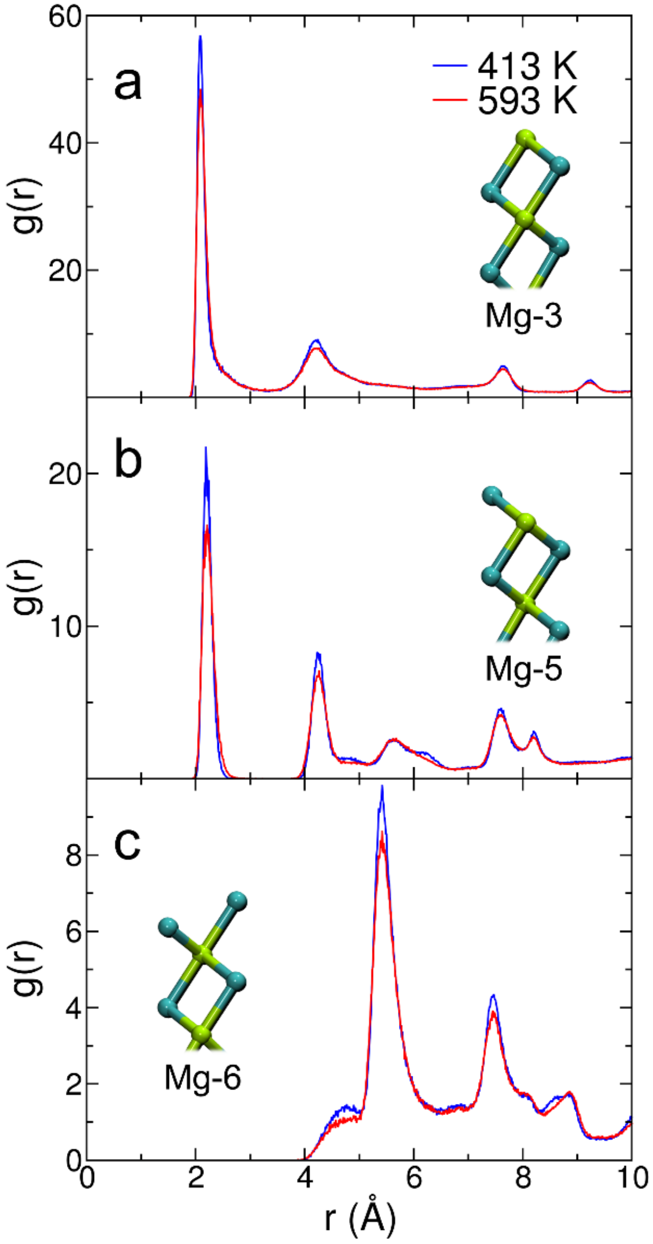

Figure S4: Radial pair distribution functions *g(r)'s* calculated between the oxygen atom of the water molecules and the magnesium cations on the surface of the Mg-3 (a), Mg-5 (b), and Mg-6 (c) layers from the MD simulations of the water/$MgCl_2$(100) system at 413 and 593 K.

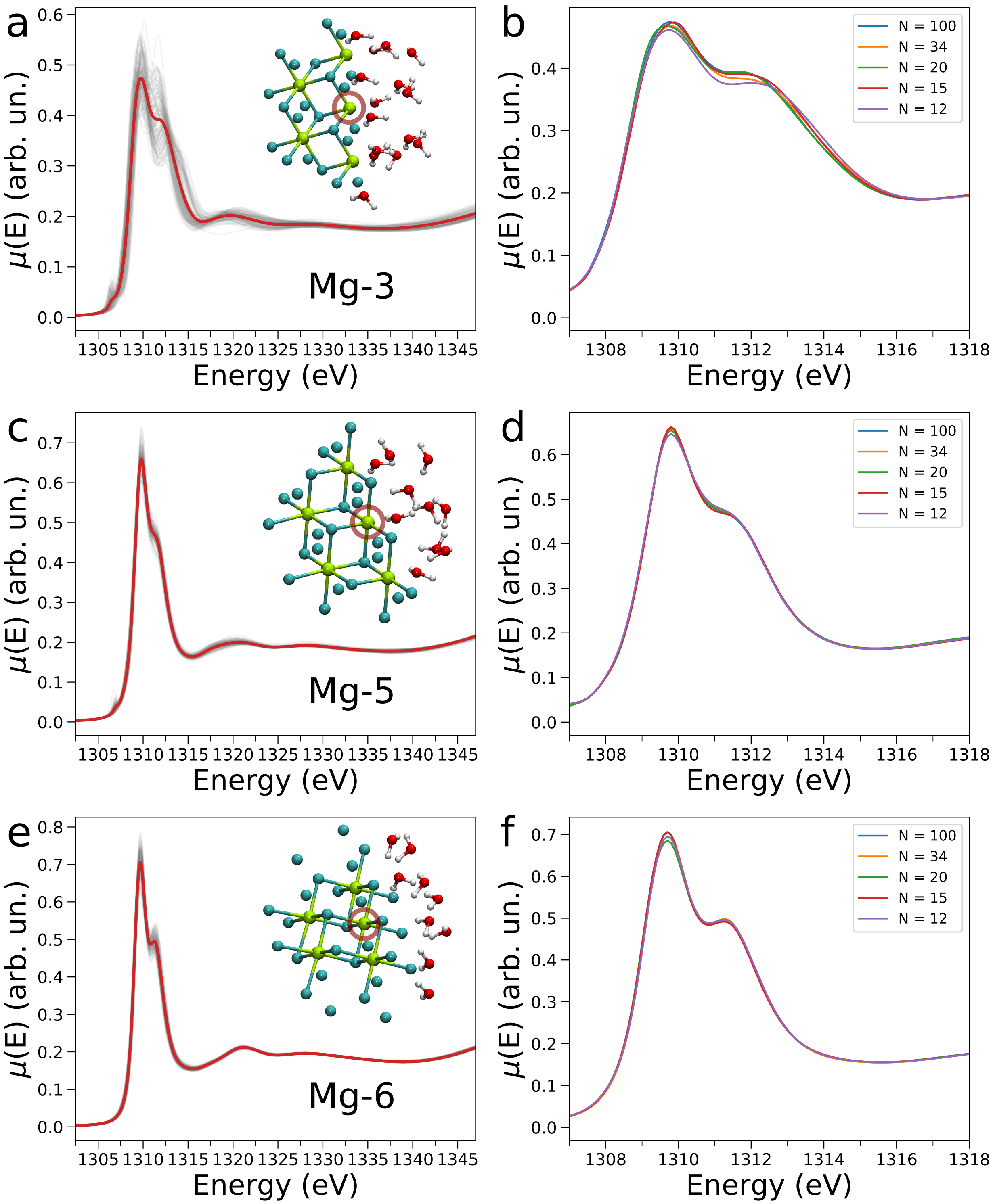

Figure S5: Theoretical NEXAFS spectra (grey lines) of the Mg-3, Mg-5 and Mg-6 sites (panels a, c and e, respectively) calculated from 100 MD snapshots of the water/$MgCl_2$ interface at 593 K and corresponding NEXAFS averages (red lines) of the 100 spectra. In panels a, c and e the Mg^{2+} photoabsorbing species are evidenced with circles in the associated molecular clusters, which include all atoms within 6 Å from the photoabsorber. A selection of average NEXAFS spectra calculated with an increasing number of spectra is shown for the Mg-3, Mg-5 and Mg-6 sites in panels b, d and f, respectively (green: Mg, cyan: Cl, red: O, white: H atoms).

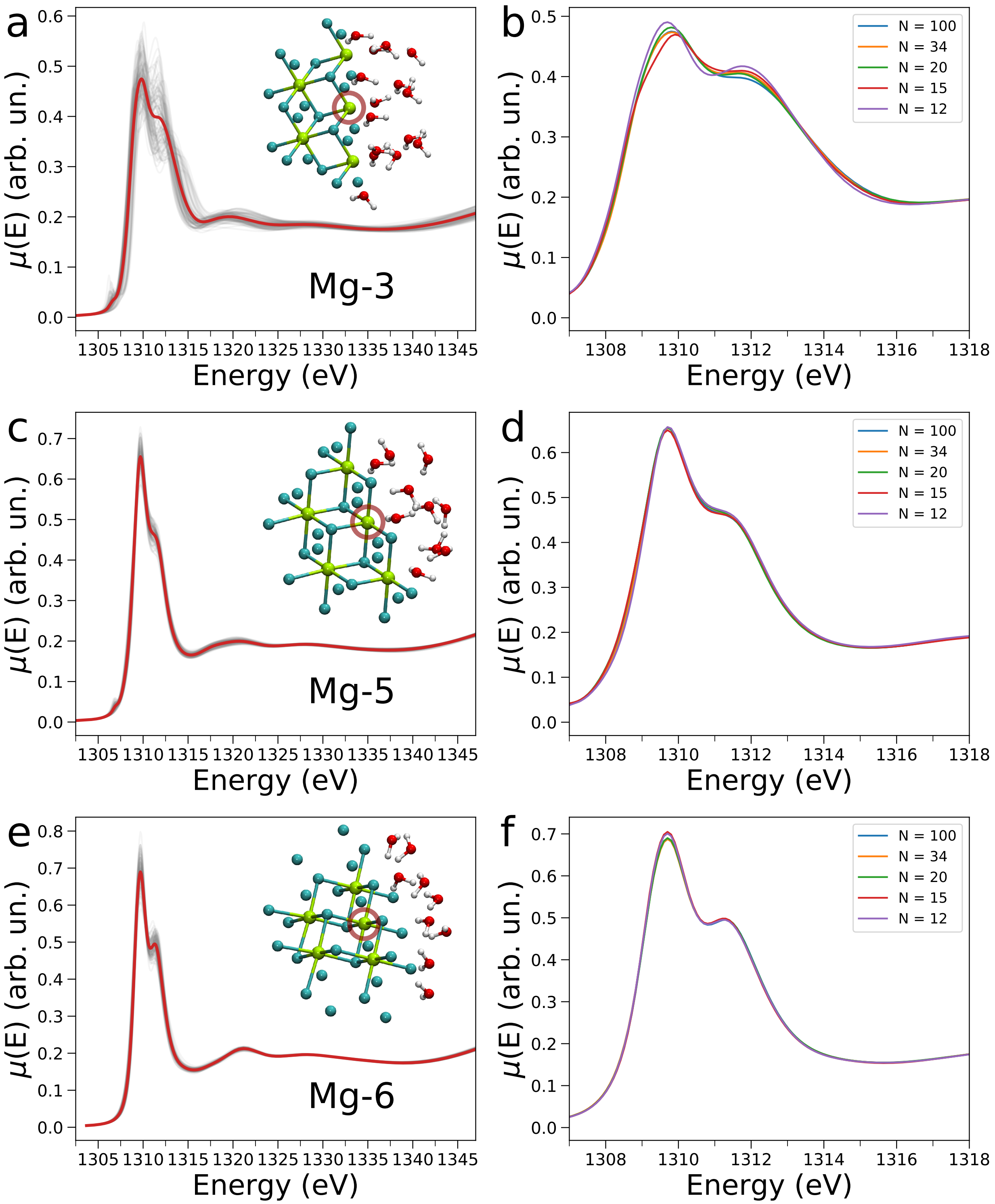

Figure S6: Theoretical NEXAFS spectra (grey lines) of the Mg-3, Mg-5 and Mg-6 sites (panels a, c and e, respectively) calculated from 100 MD snapshots of the water/$MgCl_2$ interface at 413 K and corresponding NEXAFS averages (red lines) of the 100 spectra. In panels a, c and e the Mg^{2+} photoabsorbing species are evidenced with circles in the associated molecular clusters, which include all atoms within 6 Å from the photoabsorber. A selection of average NEXAFS spectra calculated with an increasing number of spectra is shown for the Mg-3, Mg-5 and Mg-6 sites in panels b, d and f, respectively (green: Mg, cyan: Cl, red: O, white: H atoms).

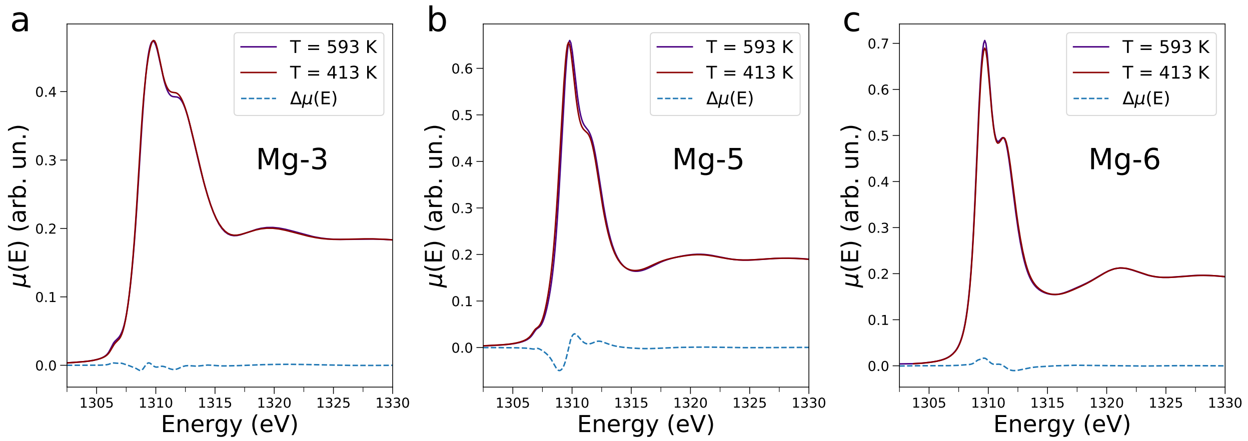

Figure S7: Comparison of the theoretical NEXAFS Mg K–edge spectra resulting from the average of 100 snapshots of MD simulations performed at 593 and 413 K (purple and red lines, respectively) and evaluated for the Mg-3, Mg-5 and Mg-6 sites (panels a, b and c, respectively). The difference $\Delta\mu$(E) between each NEXAFS average pair is shown in all panels.

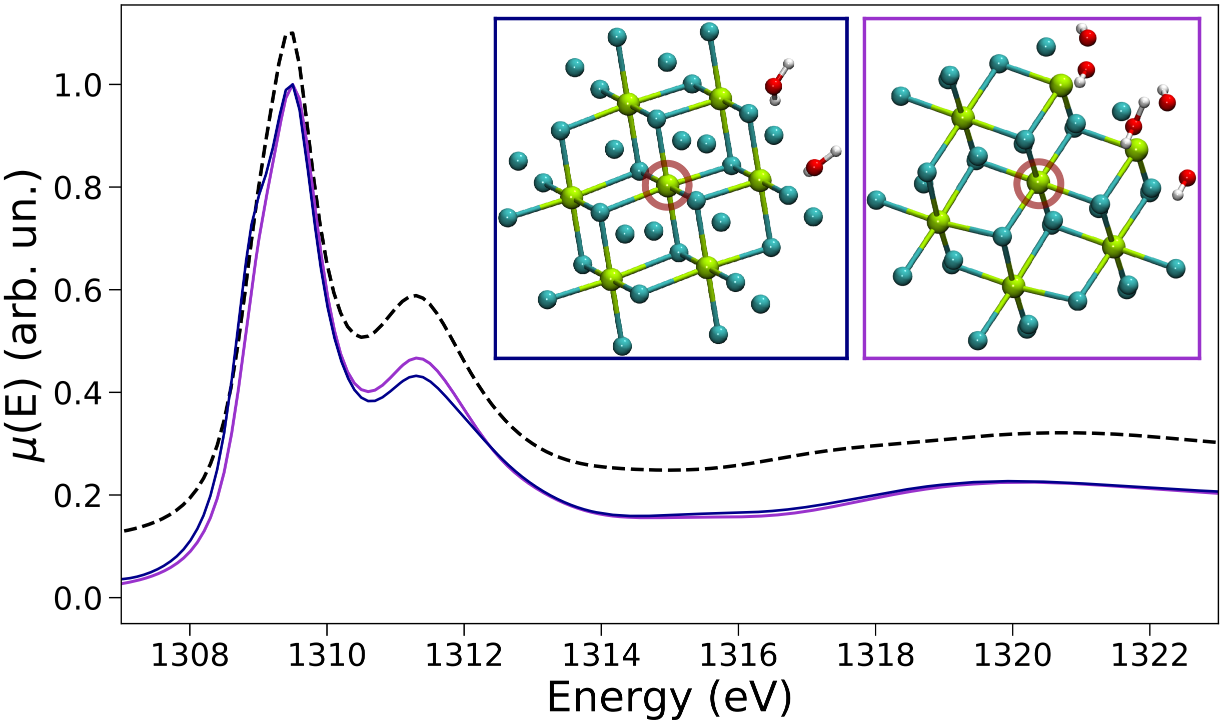

Figure S8: Comparison of the theoretical NEXAFS MD-averaged spectra evaluated for the Mg_{sub}-5 and Mg_{sub}-3 sites (blue and purple lines, respectively). The Mg^{2+} photoabsorbing species are circled in the associated molecular clusters, which include all atoms within 6 Å from the photoabsorber.

References

[1] Martini, A.; Guda, S.; Guda, A.; Smolentsev, G.; Algasov, A.; Usoltsev, O.; Soldatov, M.; Bugaev, A.; Rusalev, Y.; Lamberti, C.; Soldatov, A. *Comput. Phys. Comm.* **2019**, 107064.

[2] Joly, Y. *Phys. Rev. B* **2001**, *63*, 125120.

[3] Bunău, O.; Joly, Y. *J. Phys: Condes. Matter* **2009**, *21*, 345501.

[4] Guda, S. A.; Guda, A. A.; Soldatov, M. A.; Lomachenko, K. A.; Bugaev, A. L.; Lamberti, C.; Gawelda, W.; Bressler, C.; Smolentsev, G.; Soldatov, A. V.; Joly, Y. *J. Chem. Th. Comp.* **2015**, *11*, 4512–4521.

[5] Joly, Y.; Bunău, O.; Lorenzo, J. E.; Galéra, R. M.; Grenier, S.; Thompson, B. *Journal of Physics: Conference Series* **2009**, *190*, 012007.

[6] Rankine, C. D.; Madkhali, M. M. M.; Penfold, T. J. *J. Phys. Chem. A* **2020**, *124*, 4263–4270.
